# Material Variability and Quality Control Effects on Shear Resistance of RC Structures: A Reliability Sensitivity Study

**DOI:** 10.3390/ma19102133

**Published:** 2026-05-19

**Authors:** Saeideh Faghfouri, Alfred Strauss

**Affiliations:** Institute of Structural Engineering, Department of Landscape, Water and Infrastructure, University of Natural Resources and Life Sciences, Peter-Jordan-Straße 82, 1190 Vienna, Austria; alfred.strauss@boku.ac.at

**Keywords:** probabilistic framework, material variability, local reliability assessment, reliability analysis, sensitivity analysis, reliability index, LHS, FORM

## Abstract

The reliability of engineering structures is essential to ensure safety, durability, and sustainability. In reinforced concrete (RC), shear resistance is one of the most uncertain design aspects due to the natural variability of material properties and construction quality. Conventional design methods defined by Eurocode rely on characteristic values and partial safety factors that may not reflect the actual performance of in situ concrete. This study proposes a probabilistic framework for shear assessment that integrates material variability derived from conformity testing. Statistical parameters, including mean value and coefficients of variation (COV) of compressive strength, are incorporated into comparative reliability analysis using the First-Order Reliability Method (FORM) and Latin Hypercube Sampling (LHS). Parametric analyses are performed to quantify the influence of material variability on the reliability index *β* and failure probability $P_{f}$. The effect of varying the coefficient of variation (CoV) of the concrete compressive strength is investigated in the range from 0.01 to 0.2, both under the assumption of statistical independence and with consideration of correlation between selected variables. The sensitivity analysis is carried out to provide clear insight into the influence of uncertainty in the input parameters on the reliability of the considered limit state. The proposed framework provides a more realistic representation of structural safety and supports data-driven, performance-based management of concrete infrastructures.

## 1. Introduction

The extension of the service life for existing concrete infrastructure represents a major challenge in modern structural engineering. Many reinforced concrete structures were designed using deterministic approaches that do not explicitly account for uncertainties in material properties, loads, and degradation processes. As infrastructure ages and inspection data become increasingly available, there is a growing need to transition toward probabilistic and reliability-based assessment methods.

Shear resistance in reinforced concrete structures is particularly critical due to its sensitivity to load variability, material variability, and modelling uncertainties. Previous studies have investigated reliability-based shear behavior in various contexts, including fiber-reinforced systems, FRP-strengthened (fiber reinforced polymer/plastic) elements, and punching shear mechanism. Liao et al. [1] investigated the reliability-based shear capacity model of steel reinforced engineered cementitious composites (ECC) beams provided by fibers for ensuring the structural shear safety. Huang et al. [2] evaluated the reliability level of design guidelines for concrete structures shear strengthened with fiber-reinforced polymer (FRP). Elgohary, H.A. [3] studied the reliability of ACI-318-19, Eurocode 2 (EC-2), and Russian Code SP-63.13330 punching shear design calculations for edge column-flat slab connections without shear reinforcement. However, most studies rely on assumed statistical parameters rather than measured data from real structures. Furthermore, current design provisions in EN 1992-1-1 [4] follow a semi-probabilistic approach based on characteristic values and partial safety factors. While suitable for new design, this approach does not allow direct evaluation of the actual reliability of existing structures where material properties can be measured [5,6].

This work addresses this gap by integrating conformity test data into a probabilistic reliability framework. This study presents a probabilistic assessment of the shear resistance of reinforced concrete tunnel lining segments without transverse reinforcement, considered as a representative case of critical concrete infrastructure. The conventional deterministic design approach is reformulated within a reliability-based framework to enable a quantitative evaluation of structural safety.

The primary objective is to investigate the influence of material variability derived from conformity testing, uncertainties in input parameters, and statistical as well as functional dependencies between variables on the resulting reliability level. By integrating these aspects into the probabilistic model, this study provides deeper insight into the governing mechanisms affecting the safety of existing concrete structures and highlights the importance of data-driven approaches for performance-based assessment [5,6,7,8,9].

## 2. Limitation and Scope of This Study

It should be emphasized that this study presents only a part of the broader project on the development of calculation tools for global system probabilistic verification [10]. Due to the focus on local conditions, the results are, therefore, subject to several limitations related to the modelling assumptions and the defined scope. First, the analysis is conducted at the cross-section level and does not account for system-level reliability effects or load redistribution within the tunnel lining, which is inherently a spatial structural system. Therefore, the results should be interpreted as a local reliability assessment rather than global safety evaluation. Second, only single failure mode, namely shear resistance, without transverse reinforcement according to EN 1992-1-1 [4], is considered. Other relevant limit states, such as bending, axial failure, or serviceability criteria, are not included.

Furthermore, model uncertainty is not explicitly quantified, which may influence the absolute values of the reliability index. In addition, the analysis is time-invariant, and deterioration mechanisms such as carbonation, reinforcement corrosion, cracking, and long-term strength degradation are not considered. These processes can significantly affect the reliability of concrete structures over their service life.

Despite these limitations, this study provides valuable insights into the influence of material variability and uncertainty on structural reliability considering statistical and functional dependencies between variables and correlation. The identified trends particularly the transition from load-controlled to material-controlled behavior with increasing coefficient of variation remain robust and are expected to be valid even in more advanced modelling frameworks.

Future work should extend the present approach toward system-level reliability analysis, durability design, incorporate time-dependent deterioration models (corrosion, cracking), and integrate monitoring and inspection data [5,6,7,8,9] to enable a comprehensive, life-cycle-based reliability assessment of concrete infrastructure.

## 3. Theoretical Concept

The aim of this section is to outline the theoretical framework for the statistical analysis and to provide an overview of the simulation methods employed, including Monte Carlo simulation, Latin Hypercube Sampling, and First-Order Reliability Method (FORM).

### 3.1. Reliability Theory

**Figure 1:**
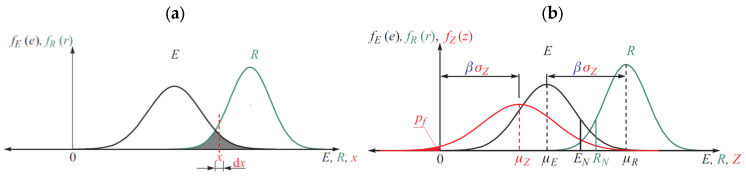
Normal distribution of safety margin as a substruction of two normal random variables and the meaning of the safety index *β*. (**a**) represented by the probability density functions $f_{E}(e)$, and $f_{R}(r)$; (**b**) Additionally represented by the safety margin and the probability density functions $f_{Z}\left(z\right)$ [11,12].

The aim of statistical and reliability analysis is mainly the estimation of statistical parameters of structural response and/or theoretical failure probability. In general, structural design consists of proportioning the elements of structure such that it satisfies various criteria of safety, serviceability, and durability under the action of loads. In other words, the structure should be designed such that it has a higher strength or resistance than the effect caused by the loads. Schematic representation of failure probability evaluation is shown in Figure 1 by considering two variables (one relating to the load E on the structure and the other to the resistance R of the structure). Both E and R are random in nature; their randomness is characterized by the corresponding probability density functions $f_{E}(e)$ and $f_{R}(r)$, respectively. Figure 1 also identifies the deterministic (nominal) values of these parameters $E_{N}\;$ and $R_{N}$ used in conventional safety factor-based approaches. The area of overlap between the two curves (the shaded region) provides basis for a qualitative measure of the probability of failure. As the distance between the two curves increases, the probability of failure decreases. The position of the curves may be represented by the means ($\mu_{E}$ and $\mu_{R}$) of the two variables. If the two curves are narrow, then the area of and the probability of failure are small. The dispersion may be characterized by the standard deviations ($\sigma_{E}$ and $\sigma_{R}$) of the two variables. The shapes are represented by the probability density functions $f_{E}(e)$*,* and $f_{R}(r)$ [11,12].

The classical reliability theory introduced the basic concept of structural reliability more formally in the form of a response variable or safety margin (in case that the function expresses failure condition) as the function of basic random variables ${X=X}_{1},X_{2},\;\ldots ,X_{N_{V}}$:

(1) $$Z=g(X)=g\left(X_{1},X_{2},\;\ldots ,X_{N_{V}}\right)$$

where g (X) (computational model) represents the functional relationship between the elements of vector X. Elements of vector X are geometrical and material parameters, load, environmental factors, etc., generally uncertainties (random variables or random fields). These quantities can be also statistically correlated.

In the case that Z is safety margin, g(.) is called the limit state function or performance function and can be formulated usually using comparison of a real load and failure load.

The structure is considered to be safe if:


(2) $$g(X)=g\left(X_{1},X_{2},\;\ldots ,X_{N_{V}}\right)\geq 0$$

The performance of the system and its components is described considering a number of limit states. A limit state function can be an explicit or implicit function of basic random variables, and it can be in a simple or rather complicated form. Usually, the convention is made that it takes a negative value if a failure event occurs. Therefore, the failure event is defined as the space where Z ≤ 0 and survival event is defined as the space where Z ≥ 0. The primary goal of the statistical analysis is the estimation of basic statistical parameters/moments of response variable Z, e.g., mean values and variances. Also, a histogram and an empirical cumulative probability distribution function are always valuable information. It can easily be done by Monte Carlo simulation, by repetitive calculations of the computational model g(.) [11,12].

Reliability analysis methods employing a reliability index or safety index take into account second moment statistics (means and variance) of the random variables. Cornell [11,12] suggested to use the distance from the expectation of the limit state function to the limit state function itself as an elementary reliability measure which consider normal PDF. This yields the reliability index:

(3) $$\beta =\frac{\mu_{Z}}{\sigma_{Z}}$$

where $\mu_{Z}$ and $\sigma_{Z}$ are the mean value and the standard deviation of the safety margin Z. In this case, *β* is actually the reciprocal value of the coefficient of variation of the variable Z.

**Figure 2:**
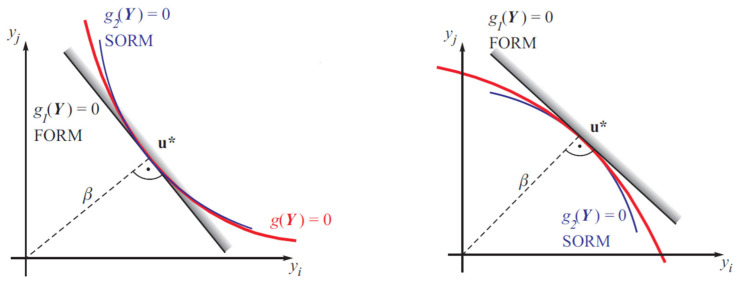
FORM and SORM methods for reliability estimation [11,12].

The reliability index can be interpreted geometrically as the minimum distance from the limit state function g(X) to the origin, see Figure 1. Hasofer and Lind [13] used this idea for generalized definition of the reliability index. They proposed using the minimum distance from limit state function (usually non-linear) to the origin in the uncorrelated normalized space as reliability measure [11,12]. Such generalized reliability index is given by:

(4) β=g(x)=0min uTu=g(x)=0min (∑i=1Nvui2)

where *β* is the distance in a standard normal space *U*-space of basic random variables and g(X) = 0 is the limit state surface. The point $u^{*}$ at which *β* reaches the minimum is called the design point. The geometric definition of *β* is symbolically illustrated in Figure 2 [11,12].

The reliability index represents the reliability measure to express reliability. Estimation of Cornells reliability index is rather simple, as it needs the estimation of basic statistical characteristics of safety margin. This task can be solved using Monte Carlo-type simulation and will be described in Section 3.2.

### 3.2. Monte Carlo Simulation, Latin Hypercube Sampling, and FORM

The FORM, Monte Carlo, and LHS methods are effective structural reliability approaches that enables the computation of a failure probability $p_{f}$ of a structural component with respect to a limit state condition g(.).

The Monte Carlo simulation technique is a well-known and widely used tool in the structural reliability analysis. Basically, it consists of N simulations (or repetitions, runs) of response or limit state function evaluations with different trials of input vector X. A special type of numerical probabilistic simulation called Latin hypercube sampling (LHS) makes it possible to use only a small number of Monte Carlo simulations. LHS is a special type of Monte Carlo numerical simulation, which uses the stratification of the theoretical probability distribution function of input random variables. The LHS is very efficient for the estimation of the first two or three statistical moments of structural response. It requires a relatively small number of simulations—repetitive calculations of the structural response resulting from adopted computational model (tens or hundreds). The utilization of the LHS strategy in reliability analysis can be rather extensive [11,12].

The main reasons for selection of LHS can be summarized as follows:

- Efficiency: good accuracy in statistical characteristics of structural response using small number of samples;
- Simplicity: the technique is suitable for implementation into complex commercial software as it requires minor modifications of program core;
- Transparency: as it represents an alternative to Monte Carlo simulation, the method is transparent and understandable also for people who are not experts in reliability engineering; generally, the Monte Carlo-type approach is close to engineering thinking.

The Latin hypercube sampling (LHS) simulation technique belongs to the category of advanced simulation method [11,12]. It is a special type of Monte Carlo numerical simulation which uses the stratification of the theoretical probability distribution function of input random variables.

The First-Order Reliability Method (FORM) has initially been proposed by Hasofer and Lind (1974) [13]. In the FORM, a linear approximation of the limit state surface in the uncorrelated standardized Gaussian space is used to estimate the probability of failure [11,12].

For this purpose, it is necessary to transform the basic variables into uncorrelated standard Gaussian variables (*U*-space):

(5) $$Y_{i}=\frac{X_{i}-\mu_{i}}{\sigma_{i}},\;i=\;1,\ldots ,N_{V}$$

where $\mu_{i}$ and $\sigma_{i}$ are the mean value and standard deviation of the random variable $X_{i}$, respectively. The reliability integral can be written in the transformed *U*-space as:

(6) $$p_{f}=\int_{g\left(Y\right)\leq 0}f_{Y}\;\left(\;Y_{1},Y_{2},\;\ldots ,Y_{N_{V}}\right){dY}_{1},{dY}_{2},\ldots {dY}_{NV}$$

The distance from the design point of the transformed limit state function to the origin is called reliability index *β*. Note that the design point is the point on the limit state surface with the minimum distance to the origin in standard normal space is considered to be important. It is also the point of maximum likelihood if the basic variables are normally distributed. This point can be obtained by solving the optimization problem expressed in Equation (4). The maximum *β* is known as the reliability index. It can be shown that the probability of failure is approximately given by:

(7) $$p_{f}=p\left(g\leq 0\right)=\phi (-\beta )$$

where *Φ* denotes the standardized Gaussian distribution function. In case of linear limit state function and normally distributed basic variables no transformations are necessary, and Equation (7) yields just the exact failure probability [11,12].

## 4. Methodology

### 4.1. General Approach

In this work, the design of shear resistance in reinforced concrete (RC) slabs is considered based on EN 1992-1-1 section 6.2 [4]. A simplified tunnel lining cross-section is considered in which geometry, actions, and material properties are treated as stochastic variables. A probabilistic framework is adopted to assess shear safety, and the analysis is conducted at the cross-section level for section of tunnel shell, focusing on shear resistance without transverse reinforcement. This approach is appropriate for identifying local critical conditions, although it does not fully represent the global behavior of the tunnel system. All input parameters appearing in the deterministic shear Equations of the referenced codes; concrete compressive strength, geometrical dimensions, permanent and variable loads, as well as model uncertainties are treated as random variables and defined by a mean value, the corresponding probability distribution, and the coefficient of variation (COV) based on the JCSS Model Code [14,15]. Based on the applied actions and material properties, the limit state function for shear failure is formulated and reliability is evaluated using **F**irst-**O**rder **R**eliability **M**ethods (FORM) and **L**atin **H**ypercube **S**ampling (LHS) simulation implemented in FReET (**F**easible **Re**liable **E**ngineering **T**ool) [11,12,16]. The investigated methodology and limit states considered in this research represent a contribution toward the completion of the development of calculation tools for global system probabilistic verification [10].

In this probabilistic framework, no partial safety factors from design standards are applied, since the verification is performed using a stochastic reliability approach rather than a conventional semi-probabilistic format. The objective is to evaluate the shear safety level of new and existing concrete infrastructure on the basis of realistic material data obtained from conformity testing and investigations.

**Figure 3:**
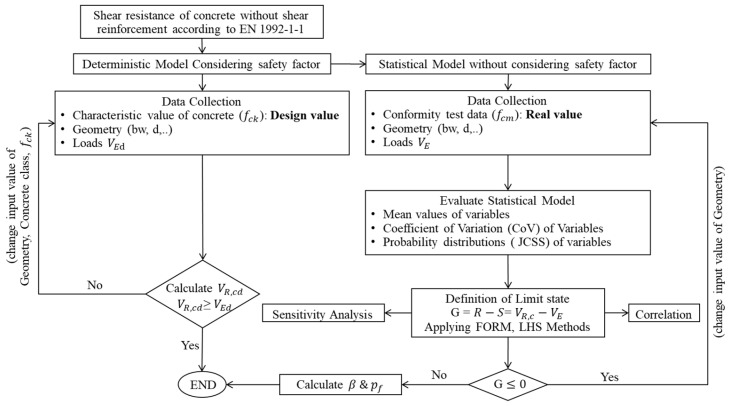
Transition from deterministic to a probabilistic reliability-based design.

The flowchart presented in Figure 3 depicts the overall methodological framework of this study. It clearly illustrates the transition from a simplified deterministic design approach to a probabilistic reliability-based methodology. Furthermore, it provides a structured comparison between deterministic and probabilistic approaches, emphasizing the enhanced capability of the latter to account for uncertainties, variability, and dependencies in structural assessment.

### 4.2. The Shear Resistance of Concrete Without Shear Reinforcement According to EN 1992-1-1

According to General Overview of EN 1992-1-1 [4] section 6.2, a minimum shear $V_{Rd,c,min}\;$ is defined to ensure a basic level of robustness independent of calculated parameters:

(8) $$V_{Rd,c}(X)>V_{Rd,c,min}(X)$$

(9) $$V_{Rd,c,min}=\left(v_{min}+k_{1}\cdot \sigma_{cp}\right)\cdot b_{w}\cdot d$$

(10) $$V_{Rd,c}=\left[C_{Rd,c}\cdot k\cdot (100\cdot \rho_{l}\cdot f_{ck}{)}^{1/3}+k_{1}\cdot \sigma_{cp}\right]\cdot b_{w}\cdot d$$

where $V_{Rd,c}$ is the shear resistance of concrete without shear reinforcement.

With: $coefficient\;k=1+\sqrt{\frac{200}{d}}\leq 2.0$, *d*: effective height in mm; coefficient $k_{1}=0.0525$ for d≤60 cm; degree of longitudinal reinforcement $\rho_{l}=\frac{A_{sl}}{b_{w}d}\leq 0.02$; the area of the tensile reinforcement $A_{sl}=\frac{\pi *\phi^{2}}{4}*n$; $b_{w}$: the smallest width of the cross-section in the tensile area [mm]; shear capacity coefficient: $C_{Rd,c}=\frac{0.15}{\gamma_{c}},\;\gamma_{c}:safety\;factor$; $\sigma_{cp}=\frac{N_{Ed}}{A_{c}}<0.2$; characteristic compressive strength $f_{ck}$ [MPa]; $N_{Ed}:$ the axial force in the cross-section due to loading or prestressing [N] ($N_{Ed}>0$ for compression) and $A_{c}$: the area of concrete cross section. This condition represents a normative requirement ensuring that the structural member fulfils the minimum safety provisions of EN 1992 1-1 [4], even if the calculated resistance based on material parameters becomes small due to unfavorable combinations of stochastic variables.

According to EN 1992-1-1 [4] section 6.2.1.3 in regions where:


(11) $${v_{Ed}\leq v}_{Rd,c}$$

No calculated shear reinforcement is necessary, where $v_{Ed}$ is the design shear force in the section considered resulting from external loading and pretesting (bonded or unbonded). In this study, the Equation (11) is considered to define limit state and statistical modelling of shear resistance.

### 4.3. Statistical Modelling and Limit Sate Implementation in FReET; Resistance Versus Action

To facilitate the interpretation of Figure 2, and as discussed in Section 3, let us assume that the limit state function g(.) is defined in terms of the resistance R and the load effect E such as g (R, E) = R − E. The probability distributions of R and E are denoted by $f_{R}(r)$ and $f_{E}(e)$, respectively. Both variables are characterized by the expected values $\mu_{R}$ and $\mu_{E}$ and their standard deviations $\sigma_{R}$ and $\sigma_{E}$. Considering Equations (8)–(11), the classical ultimate limit state verification in this case study is defined as:

(12) $$G\;(X)=V_{R,c\;}(X)-V_{E}(X)$$

where ${R=V}_{R,c\;}(X)$ is the shear capacity $E=V_{E\;}$ is the acting design shear force [17,18]:


(13) $$V_{E}\;=\;\frac{1}{2}\cdot \;k_{a}\cdot \gamma \cdot H^{2}$$

These expressions are implemented directly in the probabilistic model:


(14) $$G\;\left(X\right)=\;V_{R,c}\;(d,\;\;\phi ,\;\;f_{cm},\;\;b_{w},\;\;\sigma_{cp})-V_{E}\;(k_{a},\;\;\gamma ,\;\;H)>0$$

**Figure 4:**
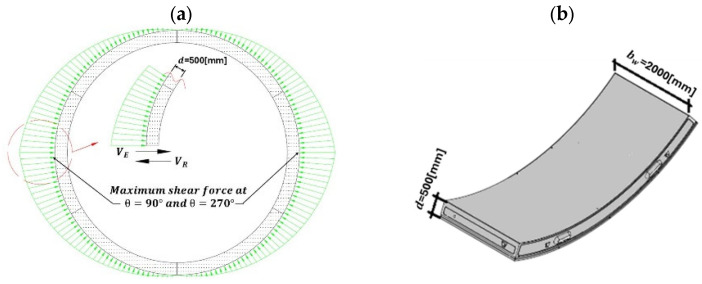
(**a**) Shear force distribution around the tunnel lining segment, (**b**) perspective view (tubbing).

G (X) represents the actual ultimate limit state against applied actions and is used to quantify the structural reliability index *β* to form the main basis for the reliability evaluation. The concrete compressive strength $f_{cm}$, diameter of reinforcement bars ϕ, minimum section width in the tensile area $b_{w}$, effective depth d, longitudinal reinforcement ratio $\rho_{l}$ and axial stress $\sigma_{cp}$, weight of the soil γ, and Overburden H are treated as stochastic variables. It should be emphasized that, in the probabilistic model and analysis, instead of using the characteristic compressive strength $f_{ck}$, the cube mean value of the concrete compressive strength obtained from the conformity test is used in the limit-state equation and for the shear resistance in the stochastic model, no partial safety factors $\gamma_{c}\;$ are applied. For the analysis, a tunnel segment cross-section [17], as shown in Figure 4, is considered and assumed to be without transverse reinforcement.

Based on the conformity test results, a mean concrete compressive strength of 70.1 MPa is adopted, which corresponds to concrete class C60/75 [19]. For this concrete class, the minimum longitudinal reinforcement for bending is calculated according to EN 1992-1-1 [4], Equation (15) and the maximum reinforcement ration using Equation (16) of the same standard:

(15) $$A_{S,min}=0.26\cdot f_{ctm}\cdot b\cdot d\;/f_{yk}$$

(16) $$\rho_{max}=\frac{A_{s}}{A_{c}}\leq 2\%$$

(17) $$a_{min}=\max\left(\phi ,\;20\;mm\;,1.2\cdot d_{korn}\right)$$

where $A_{c}$ is area of cross section $b_{w}\cdot d$. The minimum spacing $a_{min}$, i.e., the clear distance between two reinforcing bars, is also considered according to Equation (17). In this Equation, ϕ denotes the bar diameter, and $d_{korn}$ represents the maximum aggregate size of the concrete. For tunnel segments, a typical aggregate size of 0/16 mm is used. Based on these limits, the number and diameters of the longitudinal reinforcement bars used in the probabilistic model are selected. The stochastic input parameters for geometry, material properties, and actions are summarized in Table 1. The corresponding coefficients of variation and probability distribution are taken from the JCSS Model Code [14,15]. These values are then used in the reliability analysis performed with FreET 1.7.

### 4.4. Reliability Analysis in FReET

The limit state function was formulated as the difference between the shear resistance and the acting shear force. In an initial step, the reliability analysis was performed under the assumption of statistical independence among all input variables [20]. Subsequently, correlations between selected variables were introduced in order to assess their influence on the reliability index and the associated probability of failure. Reliability analyses were carried out using the mean value of cube compressive strength of concrete $f_{cm}\;$ from the conformity test. The uncertainty of the concrete strength $f_{cm}\;$ was increased from 0.01 to 0.2. The assessment procedure consists of definition of stochastic input variables from tunnel survey data, implementation of Eurocode shear model, evaluation of limit state G (X) to quantify structural safety against actions and parametric studies varying COV of mean compressive. The limit state concept allows a clear separation between code-based minimum provisions and the actual reliability of the structure, providing a sound basis for service life assessment and future durability modelling.

The reliability evaluation was performed using FORM analysis and Latin Hypercube Sampling (LHS) simulation to estimate the probability of failure $P_{f}$ and to obtain the reliability index *β* for limit state. For LHS simulation, 10,000 samples were used and for FORM analysis, a convergence criterion of $10^{-6}$ was applied. In order to ensure reproducibility and comparability of the results, the random seed is set to a fixed value for all simulations. Using a fixed seed ensures that the random numbers generated in the simulation are the same every time it is run. This allows the results to be directly comparable, because any differences in the output are caused only by changing COV of mean value of compressive strength and not by random fluctuations. Without a fixed seed, the simulation would produce slightly different results each time, making it harder to determine the true effect of the parameter changes.

The deterministic (analytical) limit state value and the results of the probabilistic analysis for $f_{cm}=70.1\;MPa$ with COV = 0.088 obtained from the conformity test are reported in Table 2 for comparison. As shown in the Table 2, the failure probability obtained from the LHS simulation in in close agreement with the analytical solution. This confirms that the probabilistic model and the sampling procedure have been implemented correctly. However, despite this agreement with the analytical solution, the failure probability obtained from LHS is significantly larger than the value predicted by FORM. This raises the question of why such a large discrepancy occurs and whether it may represent a classical reliability modelling error. To investigate this issue, an additional reliability analysis was performed with an increased overburden of 20 m. The results of this analysis are also reported in Table 2. As shown there, both the failure probability and the reliability index obtained from FORM and from LHS are now in close agreement. The reason for this behaviour is that increasing the overburden raises the stress level in the tunnel lining and moves the system from an extremely safe state with very small failure probabilities to a regime with moderate failure probability. This makes it possible to study the behaviour of FORM in two fundamentally different probabilistic regimes: one in which failure occurs only in the extreme tails of the probability distributions, and another in which failure occurs in the central region of the probability density. This comparison allows it to be determined whether the observed discrepancies originate from limitations of the reliability method or from the structural model itself.

A further important aspect is the geometric approximation of the limit-state surface in the standard normal space. In FORM, the true (generally non-linear) limit-state function is replaced by a linear tangent plane at the design point [13,21,22,23]. As a result, the value of the limit-state function at the design point, *G* (x*), computed by FORM is larger than the true value obtained from LHS or analytical evaluation. If the real limit-state surface is curved toward the safe domain, the FORM tangent plane lies above the true surface. In such cases, FORM overestimates the local safety margin *G* (x*) but underestimate the reliability index *β*.

SORM (Second-Order Reliability Method) explicitly accounts for the curvature of the limit-state surface at the design point by including second-order terms in the Taylor expansion of the limit-state function. Therefore, in problems such as the 11.5 m overburden case, where the failure domain is located in the extreme tails of skewed distributions and the limit-state surface exhibits strong curvature, SORM provides a significantly more accurate approximation of the failure probability than FORM. In contrast, when the limit-state surface is nearly planar in the relevant region, as observed for the 20 m overburden case, the difference between FORM and SORM becomes negligible, which explains the good agreement between FORM and LHS in that regime.

**Figure 5:**
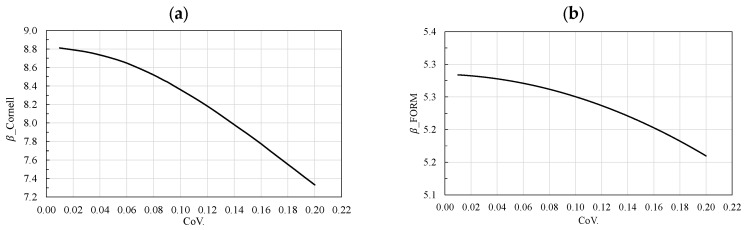
Variation of COV of mean value of cube compressive strength of concrete $f_{cm}$ vs. reliability index *β* without correlation from: (**a**) Latin Hypercube Sampling (LHS) simulation, (**b**) First Reliability Methods (FORM).

**Figure 6:**
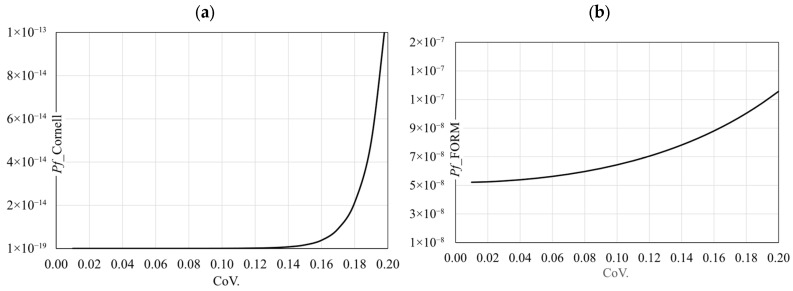
Variation of COV of mean value of cube compressive strength of concrete $f_{cm}$ vs. failure probability $p_{f}\;$ without correlation from: (**a**) Latin Hypercube Sampling (LHS) simulation, (**b**) First Reliability Methods (FORM).

As shown in Figure 5 and Figure 6, the FORM method is significantly more conservative than LHS simulation. For example, at a COV of 0.01, the reliability index obtained by FORM is approximately *β* = 5.32, whereas the LHS simulation yields *β* = 8.8. The reliability index optained by FORM is consistently lower than that obtained by LHS simulation. Although SORM would theoretically provide a more accurate estimate of the failure probability in the extreme-tail case, it was not explicitly computed here due to its higher computational cost and, as discussed in Section 3.2, the LHS simulation provides a sufficiently accurate and reliable reference for the evaluation.

As a next step, correlations between the selected variables were introduced to assess their influence on the reliability index and the associated probability of failure. Correlation describes the statistical relationship between two random variables whose values tend to vary together. In structural reliability analysis, correlations among input variables should reflect underlying physical, mechanical, or statistical dependencies. Neglecting such dependencies may lead to unrealistic combinations of basic variables and, consequently, to biased estimates of the probability of failure [21,22,23,24,25,26].

In this study, both functional dependencies and statistical correlations are explicitly considered. Functional dependencies and statistical correlations are explicitly considered. Functional dependencies arise when a variable is deterministically defined by others; for instance, the longitudinal stress is derived from the normal force and the reinforcement area. In contrast, statistical correlations describe stochastic relationships between variables that are not deterministically linked but exhibit mutual dependence due to shared influencing factors, such as material properties or geotechnical conditions.

To evaluate and compute the correlation coefficient, the following equations are used [21,22,23,24,25,26]:

(18) $$\rho_{XY}=\;\frac{Cov\;(X,\;Y)}{\sigma_{X}\sigma_{Y}}$$

(19) $$Cov\;\left(X,\;Y\right)\equiv sX,Y=\frac{1}{n-1}\;\sum \left(xi-mX\right).(yi-mY)$$

where Cov (X, Y) denotes the covariance between the variables X and Y, and $\sigma_{X},\;\sigma_{Y}$ are their respective standard deviations. The coefficient  ρ∈[−1,1] characterizes the degree of linear dependence: values of ρ>0 indicate that variables tend to increase simultaneously, whereas ρ<0 reflects an inverse relationship. In the limiting cases, ρ=1 and ρ=−1 correspond to perfect positive and negative linear dependence, respectively, while ρ=0 implies the absence of linear correlation. Within the present reliability framework, this definition is directly applied to the stochastic input variables of the limit state function $G(X)=V_{R,c}(X)-V_{E}\left(X\right)$. In particular, correlations between the reinforcement bar diameter and the longitudinal stress $\rho \left(\varphi_{s},\;\sigma_{cp}\right)$, the effective depth and the longitudinal stress $\rho \left(d,\;\sigma_{cp}\right)$, the earth pressure coefficient and the soil unit weight $\rho \left(k_{a},\gamma \right)$, as well as the effective depth and the section width $\rho \left(d,\;b_{w}\right)$, were quantified.

To quantify statistical dependencies between input variables in the reliability model, the dimensionless correlation coefficient was evaluated using MATLAB R2024a for this purpose, 200 random samples were generated for each variable within the range defined by their respective mean values, standard deviations, and coefficients of variation. Based on these, the samples’ correlation coefficients, shown in Table 3, were computed and subsequently incorporated into the joint probabilistic model. This procedure ensures that the correlation structure used in the reliability analysis is consistent with the statistical properties of the input parameters.

After introducing the correlation coefficients into the model, the analysis was repeated in the software FREET using both First-Order Reliability Method (FORM) and Latin Hypercube Sampling. However, the results obtained from the FORM-based reliability analysis with correlation were not meaningful in this case. This limitation is attributed to the strong non-linearity of the limit state function, which prevents FORM from accurately estimating the reliability index *β*.

As discussed in Section 3.2, Latin Hypercube Sampling (LHS) is more efficient and robust for this type of problem. Therefore, the subsequent reliability analysis with correlation was conducted using LHS for further investigation.

**Figure 7:**
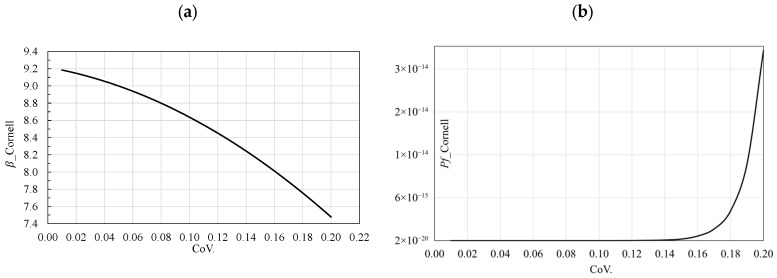
Variation of COV of mean value of cube compressive strength of concrete $f_{cm}$ (**a**) vs. reliability index *β*, (**b**) vs. failure probability $p_{f}$ obtained from Latin Hypercube Sampling (LHS) simulation with correlation.

As shown in Figure 7a, for a coefficient of variation (COV) of 0.01, the LHS simulation with correlation yields a reliability index of *β* = 9.2, while for a COV of 0.2, it results in *β* = 7.5. Figure 7b illustrates the variation of the COV of the mean cube strength versus the probability of failure $P_{F}$, providing a sufficiently accurate reference for comparison. Overall, the results indicate that the reliability analysis with correlation provides more consistent and realistic outcomes than the analysis without correlation.

In addition, a sensitivity analysis was conducted with and without correlation to identify the governing parameters. The effect of including correlation on the sensitivity results is discussed in the following section.

### 4.5. Sensitivity Analysis

Sensivity analysis enables identification of the governing variables influencing structural reliability and provides a basis for targeted design optimization, quality control, and inspection planning. The classical reliability theory introduced the basic concept of structural reliability more formally in the form of a response variable or safety margin (in case that the function expresses failure condition) and the structure is considered to be safe if [11,12,16,23]:


(20) $$Z=G\;\left(d,\;\;\phi ,\;\;f_{cm},\;\;b_{w},\;\;\sigma_{cp},\;\;k_{a},\;\;\gamma ,\;\;H\right)\geq 0$$

The main aim of reliability analysis is the estimation of reliability using probability measure called the theoretical failure probability defined as:


(21) $$p_{f}=P\;(Z\leq 0)$$

More formally, the theoretical failure probability as a measure of unreliability is definedas:

(22) $$p_{f}=\int_{D_{f}}f_{X}\;\left(X_{1},X_{2},\;\ldots ,X_{N_{V}}\right){dX}_{1},{dX}_{2},\ldots {dX}_{NV}$$

where $D_{f}$ represents failure region where g(X) ≤ 0 (integration should be performed over this region) and $f_{X}\;\left(X_{1},X_{2},\;\ldots ,X_{N_{V}}\right)$ is the joint probability density function of random variables.

Equality Z = 0 divides multidimensional space of basic random variables X = $X_{1},X_{2},\;\ldots ,X_{N_{V}}$ into safe and failure region. Explicit calculation of integral (22) is generally impossible, therefore, the application of a simulation technique Monte Carlo-type is the simple and in many cases feasible alternative to estimate failure probability integral [11,12].

The sensivity analysis, based on LHS [11,12,16] or Monte Carlo simulation [21,23], provides derivatives of the reliability index or failure probability with respect to the statistical parameters of each random variable [27]:

(23) $$S_{i}=\frac{\partial \beta }{\partial \theta_{i}}\;\;\mathrm{or}\;\;S_{i}=\frac{\partial p_{f}}{\partial \theta_{i}}$$

where $\theta_{i\;}$ represents distribution parameter of the input variables. These sensitivity factors are not normalized and, therefore, do not satisfy a unit-sum condition, in contrast to the FORM direction cosines [20,23]. By definition, the sum of squares of sensitivity factors for all random variable is equal to 1:

(24) $$\alpha_{R}^{2}+\alpha_{S}^{2}=1\;\to \sum_{i=1}^{n}{\left(\alpha_{i}\right)}^{2}=1$$

where $\alpha_{R}\;$ and $\alpha_{S}$ are the normalized sensitivity factors associated with the resistance and load effects, respectively. These coefficients define the direction of the design point in the standard normal space and quantify the relative contribution of each component to the failure mechanism.

**Figure 8:**
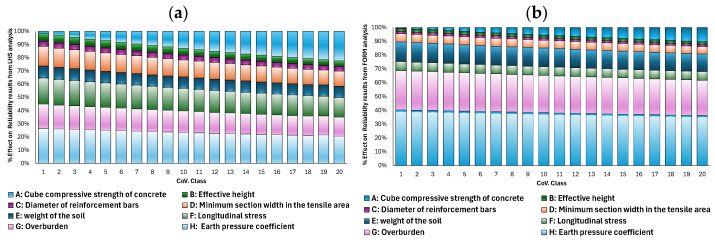
Result of sensitivity analysis without correlation (**a**) obtained using LHS method and (**b**) obtained from applying FORM method.

The results of the sensitivity analyses carried out using First-Order Reliability Method (FORM) and Latin Hypercube Sampling without correlation, as illustrated in Figure 8, provide clear insight into the influence of uncertainty in the input parameters on the reliability of the considered limit state $G(X)=V_{R,c\;}(X)-V_{E}(X)$.

In particular, the effect of varying the coefficient of variation (COV) of the concrete compressive strength was investigated in the range from 0.01 to 0.2, both under the assumption of statistical independence and with consideration of correlation between selected variables.

As shown in Figure 8b, the FORM method assigns a significantly higher contribution to the action effects compared to the LHS simulation. Consequently, these action effects exhibit a stronger influence on the resulting reliability index.

**Figure 9:**
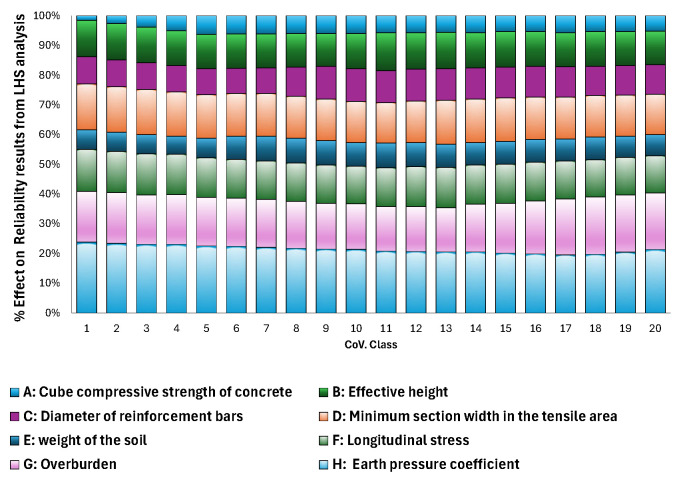
Result of sensitivity analysis with correlation obtained using LHS method.

The results of the sensitivity analysis carried out using LHS with correlation are illustrated in Figure 9.

## 5. Discussion

The results of the probabilistic analysis clearly demonstrate that the structural reliability of the tunnel lining segment subjected to shear without transverse reinforcement is highly sensitive to the uncertainty in concrete compressive strength. In particular, the coefficient of variation (CoV) of the compressive strength governs a transition in the governing failure mechanism, shifting the system response from a predominantly load-controlled regime toward a material-controlled regime.

At low levels of material uncertainty (small CoV), the reliability is primarily influenced by geometric parameters and action effects. In this range, the system behavior is relatively stable, and the variability of resistance plays a secondary role. However, as the CoV increases, the dispersion of the resistance term V_R,c_ becomes dominant. This leads to a significant reduction in the reliability index and a corresponding increase in failure probability. From an engineering perspective, this result highlights the fundamental importance of quality control of concrete production and conformity testing, since even moderate increases in variability directly translate into non-linear reductions in structural safety.

The comparison between FORM and LHS further highlights the influence of model non-linearity and probabilistic tail behavior. FORM provides an efficient and conceptually transparent estimate of the reliability index, but its accuracy is strongly dependent on the assumption of local linearity of the limit-state surface in the standard normal space. In contrast, LHS provides a more global sampling-based representation of the response surface and, therefore, captures non-linear interactions and distribution tails more robustly. The observed differences between FORM and LHS are not merely numerical discrepancies but reflect the underlying structural reality: FORM tends to be appropriate in near-linear, moderately safe regimes, whereas LHS becomes more representative when the system operates in highly non-linear or extreme probability domains.

A key outcome of this study is that LHS is particularly suitable as an evaluation and benchmarking tool for reliability assessment under uncertainty, especially when the coefficient of variation is large or when the limit state is governed by strongly non-linear resistance models such as shear capacity without transverse reinforcement. Its stratified sampling structure ensures stable estimation of statistical moments and failure probabilities with relatively efficient computational effort compared to classical Monte Carlo simulation.

The introduction of correlation between input variables significantly changes the reliability response. In the uncorrelated case, the system is governed primarily by independent contributions of material, geometric, and load variables. However, once correlation is introduced, the variables interact structurally, leading to redistribution of uncertainty influence. This results in a more physically realistic representation of structural behavior, since real concrete structures do not exhibit independent parameter variation.

The correlation analysis shows that positive correlations between geometric parameters (e.g., effective depth and section width) amplify their combined influence on resistance, while negative correlations between reinforcement area and stress reflect realistic mechanical coupling effects. These interactions reduce artificial extreme combinations that may arise in uncorrelated simulations, leading to more stable and physically consistent reliability estimates.

The sensitivity analysis provides additional insight into the governing mechanisms of uncertainty propagation. In the uncorrelated case, the sensitivity of the compressive strength increases monotonically with CoV, confirming that variability in material strength becomes the dominant driver of structural reliability. Other variables gradually lose influence, indicating a clear transition toward material-controlled failure.

In contrast, when correlation is included, the sensitivity structure becomes redistributed. Part of the influence of concrete compressive strength is transferred to correlated geometric and load-related variables. This demonstrates that sensitivity is not only a function of uncertainty magnitude but also of statistical dependence structure. Consequently, correlation does not simply modify numerical results but fundamentally changes the interpretation of which parameters govern structural reliability.

Finally, the comparison between FORM and LHS sensitivity results confirms that FORM tends to emphasize load effects more strongly, while LHS provides a more balanced representation of resistance and load contributions. This difference again reflects the local linear approximation inherent in FORM versus the global sampling nature of LHS.

## 6. Conclusions

This study presents a probabilistic reliability framework for the shear assessment of reinforced concrete tunnel lining segments without transverse reinforcement. The aim of this work was to investigate the influence of quality control on the shear resistance under local conditions. However, the definition of a truly critical reliability threshold requires consideration of the global structural system. The results show that, for a coefficient of variation between 0.1 and 0.2, the structure remains in a generally reliable state with respect to shear resistance. Nevertheless, this conclusion is case-specific and may change depending on variations in geometry, loading conditions, and structural configuration for each individual structure. The main contribution is the integration of conformity test data into a deterministic-consistent transition from stochastic to full probabilistic formulation, enabling conventional design approaches to evolve into advanced reliability-based evaluation.

Building on this foundation, this study further develops a unified deterministic–stochastic–probabilistic formulation in which traditional Eurocode-based shear design is reformulated within a stochastic framework and extended into a full probabilistic reliability model. This integrated methodology enables direct quantification of safety in terms of failure probability and reliability index, rather than relying solely on partial safety factors. A central outcome of the analysis is the dominant influence of material quality control, particularly the coefficient of variation (CoV) of concrete compressive strength. Increasing CoV leads to a pronounced non-linear reduction in the reliability index and a transition from load-controlled to material-controlled failure, clearly demonstrating that quality control and conformity testing are decisive factors for structural safety, especially in existing infrastructure where material variability is significant.

The systematic variation in CoV shows that even relatively small increases in material dispersion can substantially alter failure probability, indicating that deterministic or code-based approaches may significantly misrepresent the actual safety level if variability is not explicitly considered. Regarding computational methods, Latin Hypercube Sampling (LHS) is identified as a robust and efficient technique for reliability evaluation, offering a more global and stable representation of limit-state behaviour compared to the First-Order Reliability Method (FORM), particularly in highly non-linear problems and cases involving extreme failure probabilities. While FORM remains efficient and appropriate for moderate reliability problems with near-linear limit-state surfaces, its accuracy diminishes in the presence of strong non-linearity, extreme tail behaviour, or high uncertainty levels, where LHS—and in some cases Second-Order Reliability Methods (SORM)—provides a more reliable basis for structural assessment.

This study also highlights the critical role of correlation in reliability assessment. Correlations between input variables significantly influence both the reliability index and the sensitivity distribution by reducing unrealistic extreme combinations and introducing physically consistent dependencies among material, geometric, and geotechnical parameters. Sensitivity analysis reveals that, in the absence of correlation, concrete compressive strength increasingly dominates uncertainty propagation as CoV rises; however, when correlation is considered, this dominance is partially redistributed to other variables. This demonstrates that sensitivity results are not intrinsic properties of individual variables but depend strongly on the assumed dependency structure. Furthermore, the inclusion of correlation fundamentally alters the interpretation of system behaviour, shifting it from independent parameter contributions to a coupled network of interacting variables, thereby providing a more realistic and mechanically consistent representation of structural safety.

Overall, the findings confirm that the reliable assessment of existing concrete infrastructure cannot be achieved through purely deterministic design formats. Instead, probabilistic approaches that incorporate measured material data, statistical variability, and correlation effects are essential for realistic safety evaluation and for supporting performance-based infrastructure management.

## Figures and Tables

**Table 1:** The stochastic parameters, corresponding COV and probability distribution as input in FReET.

$G\;(X)=V_{R,c}(X)-V_{E}\left(X\right)>0$					
Notation	Variable	Unit	Distribution	Value	COV
$V_{R,c}$: Shear capacity					
$C_{R,c}$	Shear capacity coefficient *	-	-	0.15	-
*d*	Effective height	mm	Normal	500	0.01
n	Number of longitudinal reinforcement bars	-	-	40	-
ϕ	Diameter of reinforcement bars	mm	Normal	25	0.02
$f_{cm}$	Cube compressive strength of concrete	$N/{mm}^{2}$	Lognormal	70.1	0.09
$b_{w}$	Minimum section width in the tensile area	mm	Normal	2000	0.04
$\sigma_{cp}$	Longitudinal stress	$N/{mm}^{2}$	Lognormal	2	0.24
$V_{E}$: Acting shear force					
$k_{a}$	Earth pressure coefficient	-	Lognormal	0.33	0.20
γ	Weight of the soil	$N/{mm}^{3}$	Lognormal	0.000018	0.07
H	Overburden	mm	Lognormal	11500	0.07

* Without considering safety factor $\gamma_{c}$.

**Table 2:** Comparison of deterministic and probabilistic results for $f_{cm}=70.1\;MPa\;$ with COV = 0.088.

	*G*(*X*) [N]	*β*	$p_{f}$
H = 11.5 m			
Deterministic (without considering safety factor)	1,111,299	-	-
Probabilistic (FReET): Mean-LHS	1,108,000	8.5	-
Probabilistic (FReET): Mean-FORM	>1,119,500	5.3	$6.1\times 10^{-8}$
H = 20 m			
Deterministic (without considering safety factor)	316,082	-	-
Probabilistic (FReET): Mean-LHS	308,900	1	0.16
Probabilistic (FReET): Mean-FORM	>327,984	1	0.16

**Table 3:** Evaluated correlation coefficients used in the stochastic reliability analysis model.

	*d*	ϕ	$f_{cm}$	$b_{w}$	$\sigma_{cp}$	$k_{a}$	γ	H
*d*	1	0	0	0.95	−0.22	0	0	0
ϕ	0	1	0	0	−0.72	0	0	0
$f_{cm}$	0	0	1	0	0	0	0	0
$b_{w}$	0.95	0	0	1	0	0	0	0
$\sigma_{cp}$	−0.22	−0.72	0	0	1	0	0	0
$k_{a}$	0	0	0	0	0	1	−0.08	0
γ	0	0	0	0	0	−0.08	1	0
H	0	0	0	0	0	0	0	1

## Data Availability

The original contributions presented in this study are included in the article. Further inquiries can be directed to the corresponding author.
